# Efficient path routing strategy for flows with multiple priorities on scale-free networks

**DOI:** 10.1371/journal.pone.0172035

**Published:** 2017-02-15

**Authors:** Xi Zhang, Zhili Zhou, Dong Cheng

**Affiliations:** School of Management, Xi’an Jiaotong University, No. 28, Xi’an, P. R. China; Beihang University, CHINA

## Abstract

In real networks, traffic flows are different in amount as well as their priorities. However, the latter priority has rarely been examined in routing strategy studies. In this paper, a novel routing algorithm, which is based on the efficient path routing strategy (EP), is proposed to overcome network congestion problem caused by large amount of traffic flows with different priorities. In this scheme, traffic flows with different priorities are transmitted through different routing paths, which are based on EP with different parameters. Simulation results show that the traffic capacity for flows with different priorities can be enhanced by 12% with this method, compared with EP. In addition, the new method contributes to more balanced network traffic load distribution and reduces average transmission jump and delay of packets.

## Introduction

In recent years, large scale networked infrastructures, such as communication networks, power grids, and transportation networks, have come to influence many aspects of our lives. Hence, it is of great importance to investigate the structure and dynamics of such complex networks from both theoretical and practical perspective[1,2]. Since the discovery of the small-world phenomenon by Watts and Strogatz [3] and scale-free networks by Barabási and Albert [4] shed light on the structural properties of complex networks, it is more feasible to imitate real-world networks and make plausible simulations.

In terms of traffic management in real networks, it is important to design suitable strategies to improve traffic efficiency such as transportation capacity and average traveling time of packets[5–19]. However, in real-world networks, the traffic efficiency is limited due to the existence of traffic congestion. Therefore, the study on traffic congestion has received much attention in recent years [20–30]. Particularly, it has been widely revealed that both the network topology itself and the routing algorithm used in the network have great impact on the traffic congestion [31].

In order to achieve higher traffic efficiency, so called “hard” and “soft” strategies have been proposed to relieve congestion. “Hard” strategies refer to changing network topology, such as removing or adding links or nodes, so that networks can better resist congestion. On the other hand, “soft” strategies do not change network topology, instead, they try to find better traffic paths to achieve more balanced distribution for network load. In most cases, the “soft” strategies are more practicable than hard ones with high developing costs.

In the case of “soft” strategies, the shortest-path (SP) routing strategy is the most classic routing strategy[32,33], that packets are transported through paths with minimum jumps from source to destination. However, previous studies have shown that SP could easily lead to congestion on the nodes with high degree or high betweenness, in heterogeneous networks. To overcome this shortcoming, two classes of routing strategies are proposed, static routing [34–44] and dynamic routing [45–47]. Under static routing strategy, the paths for transporting packets only depend on static information of the network. The most commonly used static information is the degree of the nodes. Yan et al. proposed a routing strategy called efficient path (EP) routing strategy, where the path between source node and destination node of each packet is defined as the path in which the sum of exponent of nodes’ degree is a minimum [9]. Wang et al. proposed a local routing strategy, in which each packet is forwarded according to the probability of the neighbors’ degrees of each node [34]. In the dynamic routing strategy, packets pass through a given node or edge, depending on dynamical information, such as queue length [45], waiting time [46], or neighbors’ loads. Because traffic loads of all nodes in the network are well balanced through these routing strategies, the traffic capacity of these routing strategies can be improved several times than that of the shortest-path routing strategy. In addition, Tan F and Xia Y proposed hybrid routing on scale-free networks [48], which combines static structural properties and dynamic traffic conditions together, in order to balance the traffic between hubs and peripheral nodes more effectively. Moreover, Du WB et al. proposed a shortest- remaining- path- first queuing strategy into a network traffic model on scale-free networks, where one packet’s delivery priority is related to its current distance to the destination [49]. Although such strategy does not improve network capacity, some other indexes reflecting transportation efficiency are significantly improved in the congestion state.

Among all those “soft” strategies, the global dynamic (GD) routing strategy [19] gets the maximum traffic capacity. However, GD requires huge computation cost. On the one hand, in each time slot, queuing information of each node needs to be disseminated to all other nodes in network. On the other hand, each node needs to recalculate routing paths to all other nodes of the entire network in each time slot. Thereby, GD is rarely used in large scale network.

In real world, it is commonplace that entities have different importance levels, and require different service qualities. For example, data packets in internet are classified into time sensitive packets, such as real-time video and voice, and not time sensitive packets, such as data files for download. In the mail delivery system, mails are also classified into different types according to the payments, namely, packages with relatively higher payment needs to be delivered faster than other packets. Hence, the priority attributes needs to be considered. However, priority attributes are only discussed in [50] in recent years. In [50], flows of two levels are treated differently in two aspects. Although traffic capacity can be improved and the packets of different levels are treated differently, such strategy is even harder to be deployed than GD. In each time slot, queueing information of two levels in each node needs to be disseminated to all other nodes in network and routing paths to all nodes need to be recalculated differently for two levels.

In this paper, a novel applicable routing strategy for packets with different priorities is proposed. In our strategy, packets with different priorities are routed through different paths with different exponents of nodes’ degree in EP. Hence, this strategy is named efficient path routing with different priorities (EPWP). The performance of EPWP is investigated through simulation. First, it is shown that the traffic capacity of EPWP is about 12% larger than traditional EP without considering priorities. Second, it is revealed that network load distribution is more balanced through EPWP. Finally, the paths statistics of packets is displayed to explain how packets of different priorities are treated differently.

The rest of the paper is organized as follows. The basic model is discussed in the second section, which includes the models for the BA scale-free network and multi-priorities traffic.Then specific queuing and routing strategies are explored in the third section. Then, detailed simulation results are analyzed in the fourth section. Finally, the paper ends with the conclusion in the fifth section.

## Traffic model on scale free networks

### BA scale-free network

It has been explained that many real networks such as Internet and WWW are heterogeneous with the degree distribution following a power-law distribution *P*(*k*) ∼ *k*^−*γ*^. In this paper, the well-known Barabasi-Albert(BA) scale-free network model [4] is adopted as the physical infrastructure upon which traffic processes are taking place. BA scale-free network is generated from a fully connected graph with *m*_0_ nodes. Then, one node with *m* links is added at each step following preferential attachment that the probability Π_*i*_ of being connected to the existing node *v*_*i*_ is proportional to the degree *k*_*i*_ of *v*_*i*_, namely, Πi=ki∑jkj, where *j* runs over all existing nodes. By iterating above steps with *N*−*m*_0_ times, a BA scale-free network with *N* nodes is constructed.

### Multi-priority traffic model

Suppose that all nodes in the network are hosts and routers, which can generate and deliver packets. First, based on traditional traffic model [7], *R* packets are generated at the system in each time slot. Each packet randomly chooses source node and destination node with equal probability, as long as the source node and destination node are not the same. Second, in each time slot, each node can transport *C* packets at most. Third, each packet has the priority attribute, which is indexed by one integer *J* = 1,2,…,*N*_*prio*_, where *N*_*prio*_ denotes the total number of priorities. For each packet, the lower priority number, *J*, is, the less jump should be applied to that packet.

The distribution of priorities is as follows. Let $\left\{\gamma_{1},\ldots ,\gamma_{N_{prio}}\right\}$ be the set of priority distribution probabilities, such that 0 < *γ*_*i*_ < 1, for *i* = 1,…,*N*_*prio*_, and $\sum_{i=1}^{N_{prio}}\gamma_{i}=1$. Then, the priority of each packet, *J*, is generated as, *P*_*r*_(*J* = *j*) = *γ*_*j*_, for *j* = 1,…,*N*_*prio*_.

For simplicity, in the vast majority of the rest of the paper, the simplest case that two priorities exist with same distribution probability is considered, such that *N*_*prio*_ = 2, and $\gamma_{1}=\gamma_{2}=\frac{1}{2}$. The more complicated cases are only discussed in the last subsection of section of “Simulation Result”.

To investigate the traffic behavior under different routing strategies, the order parameter is proposed by Arenas et al to characterize the transition of traffic flow [7]:

$$H\left(R\right)=\underset{t\to \infty }{\lim}\frac{C}{R}\frac{\Delta N_{p}\left(t\right)}{\Delta t}$$

In above equation, *C* is the delivering capability of nodes, *R* is the packet generating rate, and Δ*N*_*p*_(*t*) = *N*_*p*_(*t* + Δ*t*) − *N*_*p*_(*t*) denotes the change of total number of queuing packets in the network at time, *t*. Generally, it has been found that a critical value of *R*_*C*_ exists at which a traffic phase transition occurs from free-flow state to congestion state. When *R* < *R*_*C*_, *H*(*R*) = 0 can be maintained, such that a balance can be established between newly generated packets and packets reach destination nodes. When *R* > *R*_*C*_, *H*(*R*) > 0 occurs, which leads to the accumulation of queuing packets and congestion of networks. Thus, *R*_*C*_ is a natural index to measure traffic capacity.

Moreover, in considering of the property of multi-priority, order parameter for traffic flow with priority *i* is defined as:

$$H^{\left(i\right)}\left(R\right)=\underset{t\to \infty }{\lim}\frac{C}{R}\frac{\Delta N_{p}^{\left(i\right)}\left(t\right)}{\Delta t}$$

In above equation, $\Delta N_{p}^{\left(i\right)}\left(t\right)=N_{p}^{\left(i\right)}\left(t+\Delta t\right)-N_{p}^{\left(i\right)}\left(t\right)$ denotes the change of total number of queuing packet with priority *i* in the network at time, *t*.

## Queueing and routing strategy

### Queuing strategy for multi-priority traffic model

Considering the priority attribute, traditional first-in-first-out (FIFO) queuing strategy is modified as follows. For each node, packets are buffered in *N*_*p*_ different queues, $q_{1},q_{2},\ldots ,q_{N_{p}}$, corresponding to *N*_*p*_ different priorities. For *i* = 2,…,*N*_*p*_, packets in *q*_*i*_ can be sent if and only if, *q*_1_,…,*q*_*i*−1_ are all empty queues. In each *q*_*i*_, packets are sent according to first in first out rule.

### Routing strategy for multi-priority traffic model:

In this paper, three routing strategies are compared.

Shortest path strategy (SP)[32,33].

Packets are routed to destination through paths with minimum number of jumps.

Efficient path strategy (EP)[9].

Packets are routed to destination through paths with the minimum value of:

$$L\left(P\left(i\to j\right):\beta \right)=\sum_{i=0}^{n-1}k{\left(x_{i}\right)}^{\beta }$$

In BA complex network, EP follows two properties as mentioned in [9]. First, the smaller *β* is, the smaller average paths jump value is. Second, EP obtains maximum traffic capacity, when *β* = 1. Hence, *β* is set to 1 in EP strategy in the rest of this paper.

Efficient path strategy with priority (EPWP).

Based on EP strategy, EPWP strategy is proposed in this article to cope with packets which have different priorities. Let packets in priority *i*, be routed to destination through paths with the minimum value of:

$$L\left(P\left(i\to j\right):\beta_{i}\right)=\sum_{i=0}^{n-1}k{\left(x_{i}\right)}^{\beta_{i}}$$

Since two priorities are considered throughout this article, EPWP can be denoted by two parameters, (*β*_1_,*β*_2_). Notice that EP strategy can be treated as the special case of EPWP with *β*_1_ = *β*_2_. Let *β*_2_ = 1 throughout the article, and let *β*_1_ be chosen in {0,0.1,0.2,…,0.9}. Such setting is due to the following reasons. First, packets with lower priority number should be routed through paths with relatively small jump number, thus, *β*_1_ < *β*_2_. Second, EP gets maximum traffic capacity, when *β* = 1 [9]. Hence, *β*_2_ = 1 is a good choice for transporting packets with priority, 2.

## Simulation result

### Traffic capacity simulation

The simulation of traffic capacity is carried out in BA networks with 500 nodes and 1000 nodes. Those BA networks are generated from a fully connected graph with *m*_0_ = 4 nodes, and each newly added node connects to *m* = 3 existed nodes. Results are averaged from 100 simulations on BA networks with the same parameters.

**Fig 1:**
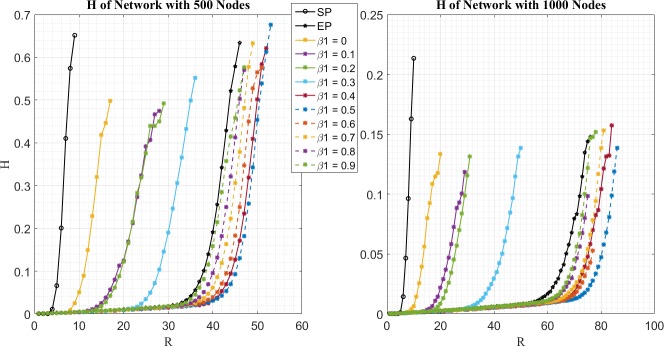
Relationship between H and R for different routing strategy on networks with 500 nodes (left figure) and 1000 nodes (right figure). Black lines with circular marker and pentagram marker denote the order parameter of SP and EP routing strategy, respectively. Lines with other colors besides black denote the order parameter of EPWP routing strategy with different value of *β*_1_.

In the first place, the results of order parameter *H*(*R*) are examined. Let *R*_*C*_ be the critical value, if *H*(*R*_*C*_)>0.1, and *H*(*R*_*C*_−1)≤0.1. Then simulation results for *H*(*R*) of different routing strategy are given by Fig 1. Following properties can be obtained.

First, among all routing strategies, SP gets the minimum *R*_*C*_. As shown in Fig 1, $R_{C}^{SP}=6$ for BA network with 500 nodes, and $R_{C}^{SP}=9$ for BA network with 1000 nodes.

Second, *R*_*C*_ of EP is much larger than that of SP. As shown in Fig 1, $R_{C}^{EP}=38$ for BA network with 500 nodes, and $R_{C}^{EP}=73$ for BA network with 1000 nodes.

Third, *R*_*C*_ of EPWP is larger than that of EP, when *β*_1_ ≥ 0.4, which means that EPWP can obtain larger traffic capacity than EP, when parameters are chosen appropriately. Moreover, EPWP can obtain the largest traffic capacity when *β*_1_ = 0.5. As shown in Fig 1, $R_{C}^{EPWP}\left(0.5\right)=46$ for BA network with 500 nodes, and $R_{C}^{EPWP}\left(0.5\right)=85$ for BA network with 1000 nodes. In general, compared with EP strategy, traffic capacity can be increased by about 12% through EPWP strategy.

**Fig 2:**
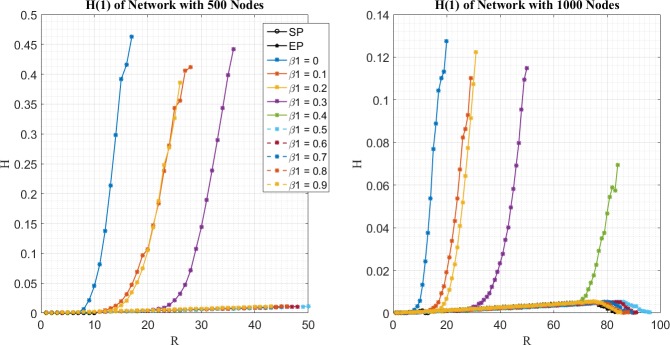
Relationship between H^(1)^ and R for different routing strategy on networks with 500 nodes (left figure) and 1000 nodes (right figure).

**Fig 3:**
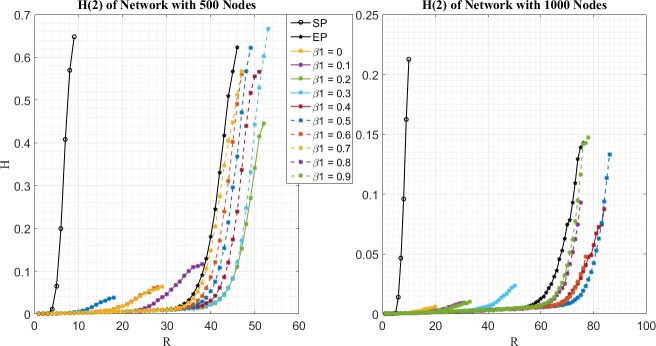
Relationship between H^(2)^ and R for different routing strategy on networks with 500 nodes (left figure) and 1000 nodes (right figure).

In the second place, simulation results for *H*^(1)^(*R*) and *H*^(2)^(*R*) of different routing strategies are given by Fig 2 and Fig 3 respectively. It can be seen that packets of two priorities are not treated homogeneously for EPWP with different parameters. As shown in Fig 2, *H*^(1)^(*R*) for EPWP with *β*_1_ < 0.4 are higher, than that for EPWP with *β*_1_ ≥ 0.4. Such phenomenon indicates that network congestion is caused by packets with priority, *i* = 1, when *β*_1_ < 0.4. On the other hand, as shown in Fig 3, *H*^(2)^(*R*) for EPWP with *β*_1_ ≥ 0.4 are higher than that for EPWP with *β*_1_ < 0.4. Such phenomenon indicates that network congestion is caused by packets with priority, *i* = 2, when *β*_1_ ≥ 0.4.

### Distribution of network load

In order to explain the reason why EPWP can improve traffic capacity, the node buffer distribution is investigated, which is defined as follows. Let *V* = {*v*_1_,…,*v*_*N*_} be the set of nodes in entire network, the degree of each node is denoted as *D*_*e*_(*v*_*i*_), for *i* = 1,…,*N*. Let *B*_*u*_(*v*_*i*_,*t*) be the buffer size of node *v*_*i*_ at time *t*. Then the average buffer size of node *v*_*i*_ is defined as:

$${\bar{B}}_{u}\left(v_{i}\right)=\underset{T\to \infty }{\lim}\frac{1}{T}\sum_{t=1}^{T}B_{u}\left(v_{i},t\right)$$

Let *V*^*j*^ be the subset of *V*, which consists of nodes whose degree is *j*, such that $V^{j}=\left\{v_{1}^{j},\ldots ,v_{\left|V^{j}\right|}^{j}\right\}\subset V$, where |*V*^*j*^| is the number of nodes in *V*^*j*^. Then, the average buffer size of nodes, whose degree is *j*, is defined as

$${\hat{B}}_{u}^{}\left(j\right)=\frac{1}{\left|V^{j}\right|}\sum_{i=1}^{\left|V^{j}\right|}{\bar{B}}_{u}\left(v_{i}\right)$$

**Fig 4:**
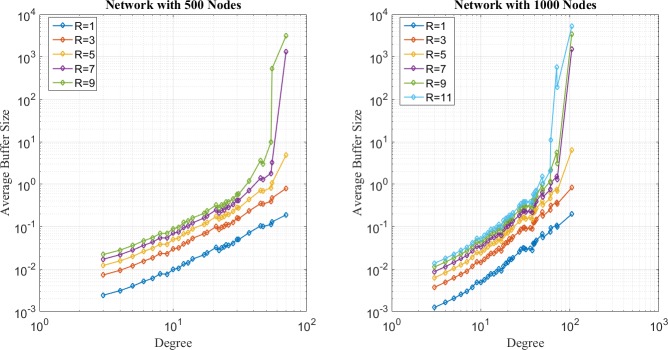
Relationship between average buffer size and node degree for SP on networks with 500 nodes (left figure) and 1000 nodes (right figure).

**Fig 5:**
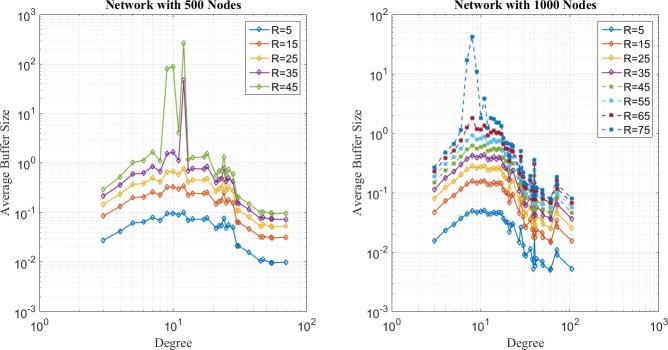
Relationship between average buffer size and node degree for EP on networks with 500 nodes (left figure) and 1000 nodes (right figure).

**Fig 6:**
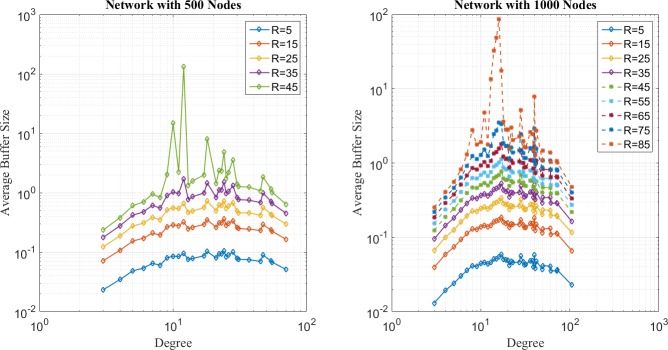
Relationship between average buffer size and node degree for EP on networks with 500 nodes (left figure) and 1000 nodes (right figure).

In the first place, distribution of network loads under SP, EP, and EPWP with *β*_1_ = 0.5, are illustrated in Fig 4, Fig 5 and Fig 6, respectively. It can be seen that distribution of network loads varies for different routing strategies.

First, network congestion can easily happen in nodes with relatively large degree for SP strategy. It can be found from Fig 4 that ${\hat{B}}_{u}^{}\left(j\right)$ increases as *j* increases. Moreover, the ${\hat{B}}_{u}^{}\left(j\right)$ is larger than 10^2^, even if *R* is less than 10, which corresponds to the results of traffic capacity simulation that SP is the worst routing strategy.

Second, the peak value of ${\hat{B}}_{u}^{}\left(j\right)$ happens in nodes with medium degree for EP strategy according to Fig 5. In other words, through EP, packets are able to avoid concentrating on those few nodes with largest degree and dispersed to those nodes with relatively lower degree. Moreover, ${\hat{B}}_{u}^{}\left(j\right)$ approximates to 10^2^, when *R* = 35 for networks with 500 nodes, and ${\hat{B}}_{u}^{}\left(j\right)$ approximates to 10^2^, when *R* = 75. Such results correspond with the results of traffic capacity simulation for EP.

Third, it can be found from Fig 6 that ${\hat{B}}_{u}^{}\left(j\right)$ has two peak values around nodes with medium degree for EPWP with *β*_1_ = 0.5, which is the best routing strategy of EPWP. Hence, compared with EP, EPWP can further disperse packets to more paths with lower degree, which results in the increase of traffic capacity. Moreover, ${\hat{B}}_{u}^{}\left(j\right)$ approximates to 10^2^, when *R* = 45, for networks with 500 nodes, and ${\hat{B}}_{u}^{}\left(j\right)$ approximates to 10^2^, when *R* = 75 for networks with 1000 nodes. Such results correspond with the results of traffic capacity simulation for EPWP with *β*_1_ = 0.5.

**Fig 7:**
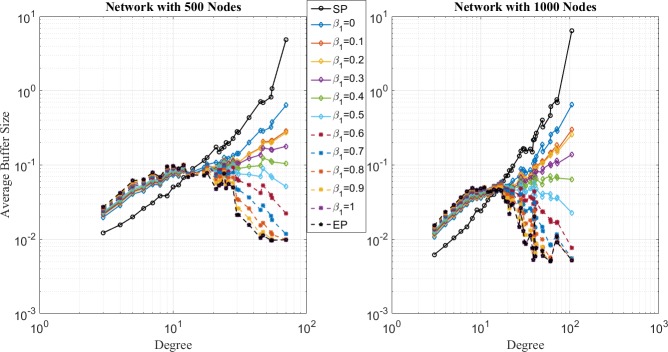
Relationship between average buffer size and node degree for different routing strategies on networks with 500 nodes (left figure) and 1000 nodes (right figure) in low traffic case.

**Fig 8:**
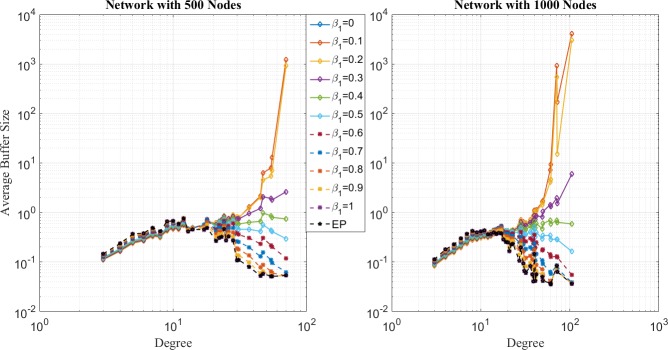
Relationship between average buffer size and node degree for different routing strategies on networks with 500 nodes (left figure) and 1000 nodes (right figure) in low traffic case.

**Fig 9:**
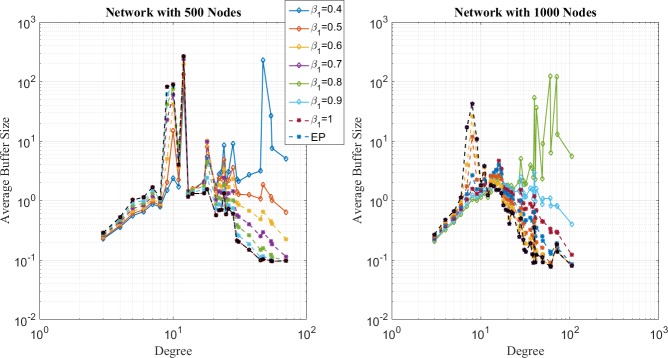
Relationship between average buffer size and node degree for different routing strategies on networks with 500 nodes (left figure) and 1000 nodes (right figure) in high traffic case.

In the second place, comparison of network loads for SP, EP and EPWP with different parameters for different traffic strength are shown in Fig 7, Fig 8 and Fig 9, which correspond to the low traffic, medium traffic and high traffic case, respectively. Following properties can be obtained.

First, it can be seen through Fig 7 that average buffer size for EPWP strategies with different parameters lies in the middle between that for EP and that for SP, under low traffic case. On the one hand, ${\hat{B}}_{u}^{}\left(j\right)$ for EPWP is smaller than that of EP, and larger than that of SP in nodes with relatively low degree. On the other hand, ${\hat{B}}_{u}^{}\left(j\right)$ for EPWP is smaller than that of SP, and larger than that of EP in nodes with relatively high degree.

Second, it can be seen through Fig 8 that, if *β*_1_ ≥ 0.4, EPWP can better balance the network load than EP does. However, if *β*_1_ < 0.4, network flow still concentrates on those few nodes with highest degree. Such phenomenon corresponds with the results of traffic capacity simulation that EPWP gets larger capacity than EP does, if *β*_1_ ≥ 0.4.

Third, it can be seen through Fig 9 that ${\hat{B}}_{u}^{}\left(j\right)$ for EPWP with different parameters have more peaks than that for EP. Hence, packets can be dispersed to more paths with EPWP than that with EP, which alleviates network congestion.

**Fig 10:**
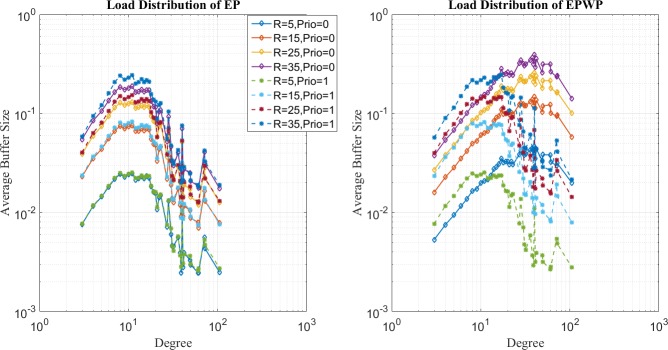
Relationship between average buffer size of two strategies and node degree for EP (left figure) and EPWP with *β*_1_ = 0.5 (right figure) on networks with 500 nodes.

**Fig 11:**
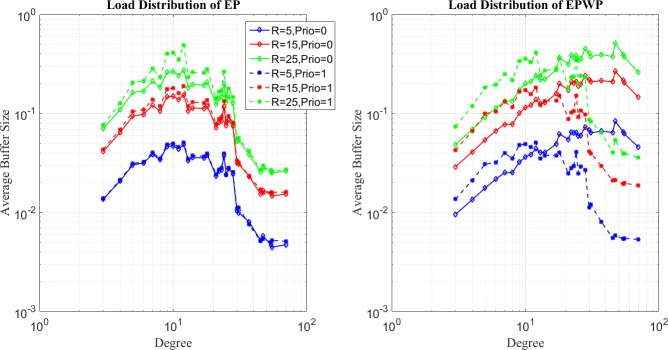
Relationship between average buffer size of two strategies and node degree for EP (left figure) and EPWP with *β*_1_ = 0.5 (right figure) on networks with 1000 nodes.

In the third place, we show how packets are dispersed through EPWP by plotting the network load distribution with different priorities. It can be seen from left figures of Fig 10 and Fig 11 that the distributions of network loads with different priorities under different traffic strength are almost the same for EP. However, for EPWP with *β*_1_ = 0.5, as shown in right figures of Fig 10 and Fig 11, node buffer distribution of packets with two different priorities are quite different. Packets with priority, *i* = 1, are more inclined to paths consist of nodes with high degree, while packets with priority, *i* = 2, are more inclined to paths consist of nodes with low degree.

### Distribution of transmission jump and delay

The transmission jump and delay distribution of packets are investigated in order to show how packets of different priorities are transported separately.

**Fig 12:**
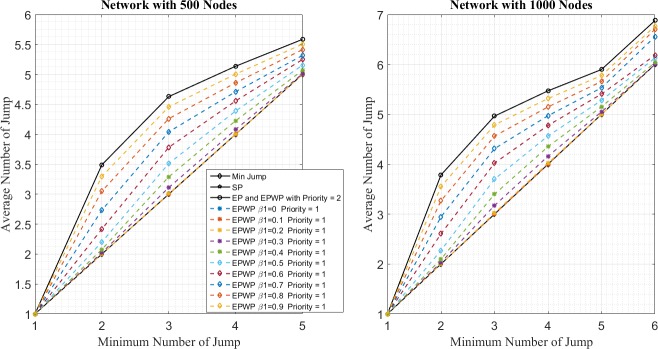
Relationship between average number of jump and minimum number of jump for different routing strategies on networks with 500 nodes (left figure) and 1000 nodes (right figure).

In the first place, Fig 12 shows the distribution of average number of jumps for packets. Let *J*^*SP*^(*n*), *J*^*EP*^(*n*), and $J_{i}^{EPWP}\left(n\right)$ be the average number of jumps for packets under SP, EP, and EPWP with priority, *i*, if the minimum number of jumps is *n*. It can be seen that the average number of jumps of packets varies for different routing strategies.

First, under SP strategy, the average number of jumps of packets equals minimum jumps, namely, *J*^*SP*^(*n*) = *n*. Second, under EPWP, packets with priority, 2, are transported through the same paths as packets transported under EP, namely, $J_{2}^{EPWP}\left(n\right)=J^{EP}\left(n\right)$. Third, it can be seen from Fig 12 that, $J_{1}^{EPWP}\left(n\right)<J^{EP}\left(n\right)$ and $J_{1}^{EPWP}\left(n\right)>J^{SP}\left(n\right)$. In other word, the average number of jumps for EPWP with priority, *i* = 1, lies between that for EP and that for SP.

**Fig 13:**
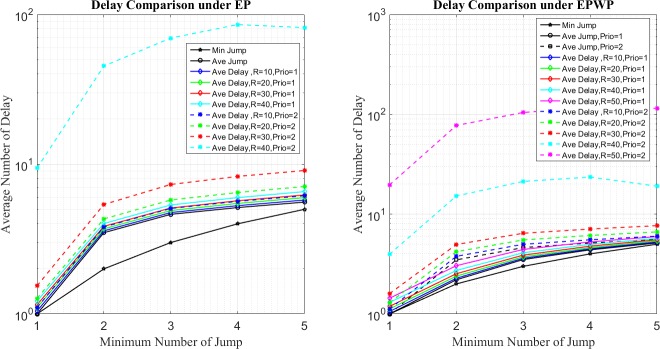
Relationship between average number of delay and minimum number of jump for EP (left figure) and EPWP with *β*_1_ = 0.5 (right figure) on networks with 500 nodes.

**Fig 14:**
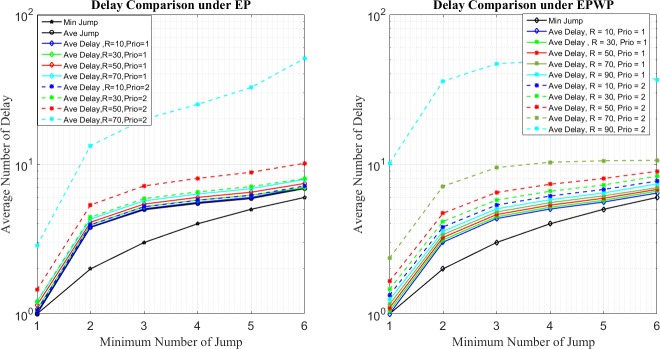
Relationship between average number of delay and minimum number of jump for EP (left figure) and EPWP with *β*_1_ = 0.5 (right figure) on networks with 1000 nodes.

In the second place, Fig 13 and Fig 14 show the delay distribution of all packets with different priorities. Let $d_{i}^{EP}\left(n,R\right)$ and $d_{i}^{EPWP}\left(n,R\right)$ be the average number of delay for packets under EP and EPWP with priority, *i*, when traffic strength is *R*.

First, it can be found from left figures of Fig 13 and Fig 14 that, $d_{1}^{EP}\left(n,R\right)<d_{2}^{EP}\left(n,R\right)$ and $d_{1}^{EPWP}\left(n,R\right)<d_{2}^{EPWP}\left(n,R\right)$ hold, no matter what *n* and *R* are. Such phenomenon is because the queuing strategy that packets with priority,1, are always sent before packets with priority, 2, in all nodes.

Second, it can be found from right figures of Fig 13 and Fig 14 that $d_{i}^{EPWP}\left(n,R\right)<d_{i}^{EP}\left(n,R\right)$ holds. Namely, under the same traffic strength, packets for EPWP can be transmitted much faster than that for EP. Such results are in accordance with the results of traffic capacity simulation, that EPWP obtains larger traffic capacity than EP does.

### Discussion of two more complicated cases

In above subsections, the simplest situation that the packets have two priorities with same distribution probabilities is discussed. In order to further exhibit the benefit of EPWP, simulation results of traffic capacity for two more complicated cases are discussed in this subsection.

The first case is the situation that packets have two priorities with different distribution probabilities. For simplicity, let *N*_*prio*_ = 2, *γ*_1_ + *γ*_2_ = 1, and *γ*_1_,*γ*_2_ ∈ {0,0.1,0.2,0.3,…,1}. Simulation results for such case are proposed in Table 1 and Table 2, which correspond to networks with 500 nodes and 1000 nodes respectively. The first row and second row denote the distribution probabilities of two priorities, respectively. The third row denote the largest values of traffic capacity for EPWP, under different distribution probabilities, when *β*_1_ is chosen in {0,0.1,0.2,…,0.9}. The fourth row denotes the optimal values of *β*_1_ for EPWP, which corresponds to the largest values of traffic capacity. Notice that when *γ*_1_ = 0 and *γ*_2_ = 1 hold, *β*_1_ doesn’t influence the traffic capacity, hence, the corresponding locations in Table 1 and Table 2 is empty. The fifth row and sixth row denote the traffic capacity for SP and EP, respectively.

**Table 1:** Simulation results for two priorities of packets with different distribution probabilities on network with 500 nodes.

Parameter	S1	S2	S3	S4	S5	S6	S7	S8	S9	S10	S11
*γ*_1_	0	0.1	0.2	0.3	0.4	0.5	0.6	0.7	0.8	0.9	1
*γ*_2_	1	0.9	0.8	0.7	0.6	0.5	0.4	0.3	0.2	0.1	0
$R_{C}^{EPWP}$	38	39	40	41	43	46	44	42	41	39	38
Optimal *β*_1_		0.2	0.2	0.3	0.5	0.5	0.5	0.7	0.9	0.9	1
$R_{C}^{SP}$	6										
$R_{C}^{EP}$	38										

**S1,S2,S3,…,S11** represent 11 scenarios with different packets distribution probabilities of two priorities. *γ*_1_ and *γ*_2_ denote the distribution probabilities of packets correspond to different priorities. $R_{C}^{EPWP}$ indicates the maximum traffic capacity of EPWP strategy for each scenario. Optimal *β*_1_ indicates the optimal value of *β*_1_ in EPWP strategy, which corresponds to the maximum traffic capacity. $R_{C}^{SP}$ and $R_{C}^{EP}$ indicate the maximum traffic capacity of SP and EP respectively.

**Table 2:** Simulation results for two priorities of packets with different distribution probabilities on network with 1000 nodes.

Parameter	S1	S2	S3	S4	S5	S6	S7	S8	S9	S10	S11
***γ***_**1**_	0	0.1	0.2	0.3	0.4	0.5	0.6	0.7	0.8	0.9	1
***γ***_**2**_	1	0.9	0.8	0.7	0.6	0.5	0.4	0.3	0.2	0.1	0
$R_{C}^{EPWP}$	73	75	79	81	84	85	83	81	78	76	73
**Optimal *β***_**1**_		0.2	0.3	0.3	0.5	0.5	0.5	0.7	0.9	0.9	1
$R_{C}^{SP}$	9										
$R_{C}^{EP}$	73										

**S1,S2,S3,…,S11** represent 11 scenarios with different packets distribution probabilities of two priorities. The meanings of *γ*_1_, *γ*_2_, $R_{C}^{EPWP}$, Optimal *β*_1_, $R_{C}^{SP}$ and $R_{C}^{EP}$ are the same as Table 1.

According to the simulation results for the first case, following properties can be found. First, the traffic capacities of EPWP are no less than that of SP and EP, under each distribution probabilities. Such property is obvious, since EP is the special case of EPWP. Second, different distribution probabilities correspond to different traffic capacities, as well as different optimal values of *β*_1_. Such property indicates that different distribution probabilities need to be treated with different parameters for EPWP, in order to increase traffic capacity.

The second case is the situation that packets have three priorities with same distribution probabilities. Hence, *N*_*prio*_ = 3 and $\gamma_{1}=\gamma_{2}=\gamma_{3}=\frac{1}{3}$, holds. Moreover, according to the description of EPWP strategy, three parameters (*β*_1_,*β*_2_,*β*_3_) are needed, which correspond to three priorities. For simplicity, let *β*_3_ = 1, *β*_1_ ≤ *β*_2_ ≤ *β*_3_ and let *β*_1_, *β*_2_ be chosen in {0,0.1,0.2,…,0.9}. Such setting is due to the following reasons. First, packets with lower priority number should be routed through paths with relatively small jump number, thus, *β*_1_ ≤ *β*_2_ ≤ *β*_3_. Second, EP gets maximum traffic capacity, when *β* = 1 [9]. Hence, *β*_3_ = 1 is a good choice for transporting packets with priority, 3. Simulation results for such case are proposed in Table 3 and Table 4, which correspond to networks with 500 nodes and 1000 nodes respectively. The first row of Table 3 and Table 4 denotes the values of *β*_1_. The second row denotes the maximum traffic capacities of EPWP for each fixed *β*_1_, when *β*_1_ ≤ *β*_2_ ≤ *β*_3_ = 1. The third rows denote the optimal values of *β*_2_, which correspond to the maximum traffic capacities of EPWP. The fourth row and fifth row denote the traffic capacities of SP and EP, respectively.

**Table 3:** Simulation results for three priorities of packets with same distribution probabilities on network with 500 nodes.

Parameter	S1	S2	S3	S4	S5	S6	S7	S8	S9	S10	S11
*β*_1_	0	0.1	0.2	0.3	0.4	0.5	0.6	0.7	0.8	0.9	1
$R_{C}^{EPWP}$	16	27	43	48	50	47	44	42	40	39	38
Optimal *β*_2_	0.7	0.7	0.6	0.6	0.5	0.5	0.8	0.9	0.9	0.9	1
$R_{C}^{SP}$	6										
$R_{C}^{EP}$	38										

**S1,S2,S3,…,S11** represent 11 scenarios with different parameters of EPWP strategy. *β*_1_ indicates the value of *β*_1_ for EPWP strategy. $R_{C}^{EPWP}$ indicates the maximum traffic capacity of EPWP strategy if value of *β*_1_ is set. Optimal *β*_2_ indicate the optimal value of *β*_2_, which corresponds to the maximum traffic capacity of EPWP strategy. $R_{C}^{SP}$ and $R_{C}^{EP}$ indicate the maximum traffic capacity of SP and EP for each scenario.

**Table 4:** Simulation results for three priorities of packets with same distribution probabilities on network with 1000 nodes.

Parameter	S1	S2	S3	S4	S5	S6	S7	S8	S9	S10	S11
*β*_1_	0	0.1	0.2	0.3	0.4	0.5	0.6	0.7	0.8	0.9	1
$R_{C}^{EPWP}$	25	49	76	86	89	87	83	79	77	75	73
Optimal *β*_2_	0.8	0.7	0.6	0.6	0.5	0.5	0.8	0.8	0.9	0.9	1
$R_{C}^{SP}$	9										
$R_{C}^{EP}$	73										

**S1,S2,S3,…,S11** represent 11 scenarios with different parameters of EPWP strategy. The meanings of *β*_1_, $R_{C}^{EPWP}$, Optimal *β*_2_, $R_{C}^{SP}$ and $R_{C}^{EP}$ are the same as Table 3.

According to the simulation results for the second case, following properties can be found. First, according to simulation results on Table 3 and Table 3, it can be found that the maximum traffic capacities of EPWP are obtained when *β*_1_ = 0.4 and *β*_2_ = 0.5. Second, the maximum traffic capacities of EPWP are 50 for networks with 500 nodes, and 89 for networks with 1000 nodes. Hence, under the situation that the packets have three priorities with same distribution probabilities, maximum traffic capacity of EPWP is still larger than that of SP and EP. Third, traffic capacity of EPWP may be smaller than that of EP, if parameters of EPWP are not set appropriately. For example, if *β*_1_ = 0, the traffic capacity of EPWP is 16 for networks with 500 nodes, and 25 for networks with 1000 nodes, which are much smaller than that of EP.

## Conclusion and future work

This paper proposes a novel routing algorithm to overcome the traffic congestion problem. In our method, packets of different priorities are transported through different routing paths based on EP with different parameters. Simulation results show that traffic capacity can be increased considerably and traffic loads distribution is more balanced. Moreover, the average transmission delays of packets are reduced compared with EP.

In the vast majority part this paper, the simplest case that the packets have two priorities with same distribution probabilities is considered and the traffic capacity of two more complicated cases are discussed. However, the general situation that packets have any number of priorities with random distribution probabilities is not discussed in this paper. Therefore, following problems need to be studied in the future. First, researchers need to figure out the optimal parameters of EPWP for the general situation in order to obtain the maximum traffic capacity. Second, it is necessary to study the distribution of network load for the general case under EPWP strategy. Based on such study, EPWP strategy might be improved.
